# Diagnostic Challenges in Affective Disorders: Delirious Mania - A Case Report and Literature Review.

**DOI:** 10.1192/j.eurpsy.2024.908

**Published:** 2024-08-27

**Authors:** P. Herrero Ortega, A. Oliva Lozano, J. Garde González, M. A. Morillas Romerosa

**Affiliations:** ^1^Psychiatry, La Paz University Hospital, Madrid, Spain

## Abstract

**Introduction:**

Affective disorders exhibit diverse clinical manifestations, and one distinctive subtype is delirious mania. Despite its exclusion from formal diagnostic manuals, delirious mania frequently emerges in everyday clinical practice. Recognizing it within the realm of differential diagnosis is crucial. Delirious mania is characterized by acute onset of excitement, grandiosity, emotional lability, delusions, and insomnia typical of mania, combined with disorientation and altered consciousness characteristic of delirium. Some authors consider delirious mania as a variant of classic bipolar disorder, while others associate it with catatonia. Additionally, some link it to underlying medical or neuropsychiatric causes.

**Objectives:**

To describe the clinical case of a patient with delirious mania and emphasize the importance of recognizing this as a potencial diagnosis in patients with abrupt alterations in mental state.

**Methods:**

Clinical case report and literature review.

**Results:**

A 61-year-old female patient with a history of a unique depressive episode over 20 years ago, treated with Carbamazepine up to 750 mg, is admitted to the Emergency Room with acute symptoms consistent in global disorientation, agressive behavior, mutism, bradyphrenic and repetitive incoherent speech, along with visual hallucinations, all of which had developed over a few days. The gradual withdrawal of Tegretol over an 8-month period preceded her admission to the ER.

Relevant medical tests, including cranial CT, EEG, blood tests, and urine analysis, were conducted during her ER stay, all of which yielded normal results. Neurological evaluation ruled out acute neurological pathology, leading to her subsequent admission to the Psychiatry department. Throughout her admission, the patient exhibited irritability and expressed derogatory comments filled with offensive language. She gradually became more expansive, with her thought content becoming megalomaniac in a delirious range. Her speech was incoherent, verbose and had loose associations.

Treatment was reintroduced with Carbamazepine up to 600 mg/day and Olanzapine up to 20 mg/day, resulting in a rapid and comprehensive improvement of her symptoms, ultimately leading to the complete resolution of her condition.

**Conclusions:**

This case highlights the concept of delirious mania, characterized by alterations in attention, orientation, memory, confusion, behavioral and thought fluctuations, and psychomotor disturbances which can manifest abruptly, as observed in this patient. This clinical case underscores the significance of considering delirious mania in the differential diagnosis of patients with abrupt alterations in mental state, particularly those of advanced age with a history of affective episodes. A global understanding of this condition is essential for its timely recognition and appropriate management.

**Disclosure of Interest:**

None Declared

